# Co-designing a place-based social and emotional wellbeing service model with young Aboriginal people in the remote Fitzroy Valley of Western Australia: the Bigiswun Kid project

**DOI:** 10.1080/00049530.2025.2538509

**Published:** 2025-08-03

**Authors:** Emily Carter, Lauren J. Rice, Emma Bear, Mudge Bedford, Cheyenne Carter, Jadnah Davies, Nikkita Rice, Sue Thomas, Fergus Wells, Elizabeth J. Elliott

**Affiliations:** a Marninwarntikura Women’s Resource Centre, Fitzroy Crossing, Western Australia; b Faculty of Medicine and Health, Specialty of Child and Adolescent Health, The University of Sydney, Sydney, New South Wales, Australia

**Keywords:** Aboriginal and Torres Strait Islander Peoples, adolescent, rural and remote, social and emotional wellbeing, mental health, fetal alcohol spectrum disorder

## Abstract

**Objectives:**

The current study aimed to work with young Aboriginal people from the very remote Fitzroy Valley in Western Australia to (1) identify their social and emotional wellbeing (SEWB) support needs, and (2) report how they would like these supports delivered.

**Methods:**

We interviewed 83% (*n* = 94) of young people aged 16–19 from the Fitzroy Valley and 89% (*n* = 101) of their parents. Using anf Aboriginal Participatory Action Approach, we piloted some SEWB supports and worked with 10 young people to co-design the SEWB service.

**Results:**

Based on the interviews and co-design consultation with young people, we identified five supports to be provided in the SEWB service. These included mental health support, particularly clinical psychology for people with moderate mental illness; community wellbeing workshops; male- and female-specific wellbeing workshops; and support to access existing services.

**Conclusions:**

Partnering with young people ensured that community strengths and challenges were incorporated in the service design. Study findings were used to secure funding for the implementation and evaluation of a SEWB service in the Fitzroy Valley. The process and lessons learned in the Project could be used to engage, consult and partner with young people to design services in other remote regions.

## KEY POINTS

What is already known about this topic:

1. Through Closing the Gap Target 14, all Australian governments have committed to improving the social and emotional well-being of Aboriginal andTorres Strait Islander People.
2. Nationally, this Target is worsening over time, with rates of suicide and self-harm particularly high in remote regions of Western Australia.
3. Place-based care models enable the strengths and complexities of remote Aboriginal communities, including culture, language, and geographical isolation, to be considered and utilised in service design, increasing the likelihood of service success.

**What this topic adds:**

1. The Fitzroy Valley has a gap in mental health care for moderate mental illness, and young people feel this is best addressed with consistent access to clinical psychology.
2. To address this gap young people proposed a potential model for telehealth psychology in remote Aboriginal communities.
3. Young people requested that staff working in SEWB services in remote Aboriginal communities be consistent, efficient, open-minded, confidential, and provide support tailored to the young people's strengths and support needs.

Aboriginal and Torres Strait Islander Peoples have continued their traditional way of life in Australia for at least 60,000 years (Clarkson et al., 2017). Some strengths of Aboriginal and Torres Strait Islander cultures include a respectful and spiritual connection with the land and environment, sophisticated protective kinship systems, and an emphasis on community, which fosters a sense of identity and belonging (Dudgeon et al., 2014; Hunter et al., 2021). For the past 250 years, colonisation, genocides and ongoing discriminatory policies have adversely affected Aboriginal and Torres Strait Islander Peoples’ culture, way of life, health and wellbeing (Milroy et al., 2014).

Through Target 14 of the 2020 National Agreement on Closing the Gap (CTG), federal, state and territory governments have agreed to take action to ensure Aboriginal and Torres Strait Islander people enjoy high levels of social and emotional wellbeing (SEWB), and the outcome measure is a sustained reduction in suicide rates. Since the CTG Targets’ inception in 2008, Western Australia (WA) has had the highest rate of suicide passings of all Aboriginal and Torres Strait Islander people in Australia (Government of Western Australia, 2024; Productivity Commission, 2024a). At 38.1 per 100,000 in 2018–2022, this suicide rate is among the highest in the world (Warwick et al., 2019). Within WA, rates of suicide and self-harm are highest in the northernmost Kimberley region (McHugh et al., 2016; McPhee et al., 2022), making it a priority area for addressing the SEWB needs of Aboriginal^1^ people.

The Australian National Strategic Framework for Aboriginal and Torres Strait Islander Peoples’ Mental Health and Social and Emotional Wellbeing, hereon referred to as the SEWB Framework, describes a SEWB model with nine guiding principles. Principle 2 recognises that “self-determination is central to the provision of Aboriginal and Torres Strait Islander health services”, and Principle 8 that “there is no single Aboriginal or Torres Strait Islander culture or group” p. 3 (Commonwealth of Australia, 2020). The SEWB Framework advocates place-based care models for rural, remote and under-serviced communities. Consistent with the Framework, the National Agreement on CTG outlines four Priority Reforms to inform and evaluate how governments improve their work with Aboriginal and Torres Strait Islander Peoples and communities. Priority Reform 1 aims to empower Aboriginal and Torres Strait Islander Peoples to share decision-making authority with governments, thereby accelerating policy and place-based progress. The National Agreement on CTG defines shared decision-making as “where a wide variety of groups of Aboriginal and Torres Strait Islander people, including women, young people, elders, and Aboriginal and Torres Strait Islander people with disability, can have their voices heard” p. 6 (Commonwealth of Australia, 2020).

Priority Reform 1 includes five priority areas, with SEWB being one (Australian Government, 2020). Place-based policymaking and care models, as well as shared decision-making, empower Aboriginal communities, including Aboriginal Community-Controlled Organisations (ACCOs), to lead or partner with government services in co-creating policy and service design. Place-based care models are crucial to the success of services in remote Aboriginal communities. They allow for the complexities specific to a region, such as geographical remoteness, culture, and language, to be considered and addressed in service design (Hart & Connolly, 2022). This approach moves away from the government’s “one-size-fits-all” approach, which has long proved ineffective for Aboriginal and Torres Strait Islander people (Cuervo et al., 2015). Place-based policymaking/developing care models require an in-depth understanding of a region. Although the need for community consultation with Aboriginal and Torres Strait Islander people has long been recognised, government efforts to properly consult and partner with Aboriginal people have fallen short (Australian Human Rights Commission, 2020; Productivity Commission, 2024b). Proper consultation and co-design in remote Aboriginal communities must be led by or with Aboriginal people.

The very remote Fitzroy Valley is located in the heart of the Kimberley. Most people (80%) are Aboriginal and belong to one of five language groups: Bunuba, Gooniyandi, Nykina, Walmajarri and Wangkatjungka (Morphy, 2010). Living on or close to Country within their language and sophisticated kinship systems has helped people maintain some of their culture, knowledge and traditions despite colonisation. The Fitzroy Valley consists of one town, Fitzroy Crossing, and 32 Aboriginal communities spread over a 400 km diameter. Since 2008, Senior Aboriginal women from the Fitzroy Valley have led place-based community initiatives and research to address the needs of their people, with a focus on children and young people (Pickard et al., 2025). For example, in 2008–2010, they conducted the Lililwan (Kimberley Kriol for all the little ones) Project to identify the needs of children, which helped secure government funding to establish an Early Childhood Centre, a Parent and Family Centre, and the Marulu team, which supports young people with complex needs (Elliott et al., 2012; Pickard et al., 2025). In 2019, they initiated the Bigiswun Kid (Kimberley Kriol for adolescent) Project to consult with young people from the Lililwan Project about the support they need to thrive in adolescence and early adulthood (Rice et al., 2022). The Lililwan cohort was chosen because they were provided with comprehensive management plans outlining a range of individualised medical and educational supports required, and 400 formal referrals were made to health or mental health services (Fitzpatrick et al., 2012). Although the Lililwan Project resulted in ACCO’s establishing new services (Pickard et al., 2025), women in the community were concerned that there had been insufficient improvement in government services, particularly for mental health, health and education, to deal with the large number of children with complex chronic needs. As a result, they were worried that some young people from the Lililwan cohort were struggling in adolescence (Rice et al., 2022). These concerns are reflected in service reviews (Dossetor et al., 2019; Salinas-Perez et al., 2020) and government inquiries (Fogliani, 2019; Legislative Council, 2024). The women wanted to focus on older adolescents (16–19 years) because they recognised a service gap for young people and a need to support them in transitioning out of school. The Bigiswun Kid Project aims to identify the needs of adolescents in remote Aboriginal communities and use these findings to inform local services, thereby improving adolescent health and wellbeing.

The present paper aims to: 1. identify the social and emotional wellbeing (SEWB) support needs of young people in the Fitzroy Valley; and 2. report how young people and their parents would like these supports delivered.

In the Supplementary Material, we describe how we piloted some supports, what we learned from these pilots, the co-design of a SEWB service building and outline strategies for engaging young people.

## Methods

### Positionality

This study was part of a 16-year partnership between Senior Aboriginal women from the Fitzroy Valley, currently represented by MWRC (E.C.), and clinician researchers (E.J.E.) from the University of Sydney [13]. The authors include Aboriginal researchers from the Fitzroy Valley (E.B., M.B., C.C., J.D., E.C.), non-Indigenous researchers (L.R., E.J.E., S.T.) and youth mentors (N.R., F.W.). Non-Indigenous staff received formal cultural awareness training and ongoing cultural supervision and guidance. All Aboriginal staff working on the Project received training in research activities, and the two full-time Aboriginal staff received additional training opportunities. For details, see (Rice et al., 2025). We employed reflexive practices to examine how our lived experiences influenced our perspectives throughout the Project.

### Project design

The Bigiswun Kid Project is a population-based study that used an Aboriginal Participatory Research Action (APAR) approach. For details about the methods, see our protocol paper (Rice et al., 2022) and a paper describing the APAR approach (Rice et al., 2025), including Aboriginal leadership and governance.

### Community consultation

We conducted an 18-month community consultation, during which we spoke to young people (*n* = 17), parents (*n* = 30), and stakeholders (*n* = 23), e.g., senior community members, local government services, and other ACCOs, about the purpose and design of the Bigiswun Kid Project. During the consultation, senior community members requested that support be provided to young people during the data collection to ensure immediate knowledge translation. We spoke with 30 parents and 17 young people to determine the type of support they would like for young people, as described below.

### Participants

We invited all young people born between 2002 and 2003 (aged 16–19 years in 2020–2022) living in the Fitzroy Valley and their parents to participate in interviews. These young people were initially identified through school records in 2008/2009 in the Lililwan Project (Fitzpatrick et al., 2017). A team of local Aboriginal researchers and community navigators informed the young people and their parents about the study and sought their consent for participation. The young people were invited to participate in an interview and access support offered as part of the research. They were informed that they could access the support without completing the interview.

### Data collection

#### Interviewers

The interviewers included a female and male Aboriginal researcher/youth mentor (E.B., M.B.) from the Fitzroy Valley, a non-Indigenous female Research Fellow (L.R., PhD), and a non-Indigenous female and male youth mentors (N.R., F.W.). The Aboriginal researchers were known to the young people. Eleven Aboriginal community navigators were also employed to provide the team with community-specific knowledge, language skills, cultural guidance, and governance. They helped locate, inform, and obtain consent from parents and young people about the study, supported them throughout the study activities if desired, and translated into traditional language or Kriol as needed. At least one navigator was engaged from each cluster of communities (based on distance from Fitzroy Crossing and proximity to each other) across the Fitzroy Valley. Interviews were conducted in a shady location in communities.

#### Interviews to identify the types of SEWB support young people require

During the consultation process, we asked 30 parents and 17 young people about the types of support they need. Four authors, E.C., L.R, M.B., and J.D, then reviewed and collated the responses into the following nine supports: youth mental health services, young men’s wellbeing workshops, young women’s wellbeing workshops, advocacy and help accessing existing services, youth hub, more opportunities for cultural and on-Country activities, more sporting activities, and child development workshops for young parents and public transport. Following this, all young people born in 2002–2003 and their parents were invited to participate in semi-structured interviews that systematically discussed the nine supports suggested during the consultation. Participants were asked whether they thought the suggested support was needed (providing a quantifiable yes or no response), how they felt it should be delivered, and if they could think of any other support required. Responses were written verbatim where possible. The young people and their parents were provided with a $50 grocery store voucher as a token of appreciation for their time in completing the interviews.

#### Identifying existing mental health services

During the consultation process, the Bigiswun Kid Project team met with service providers to map out what programs and supports are available to young people. The team used this information to determine where they could refer young people for support during the data collection process and identify gaps in support and services. The service map was monitored and updated throughout the two-and-a-half-year data collection period.

#### Piloting support in the research

Based on the support needs identified in the consultation and interviews, the Bigiswun Kid Team worked with the young people to determine the supports they could provide and pilot during the research. Extensive consultation continued throughout the Project, including during the piloting of supports. Notes from these consultations were used to develop the learnings for each support, which are described in the Supplementary Material.

#### Co-design of SEWB supports

**Figure 1.**
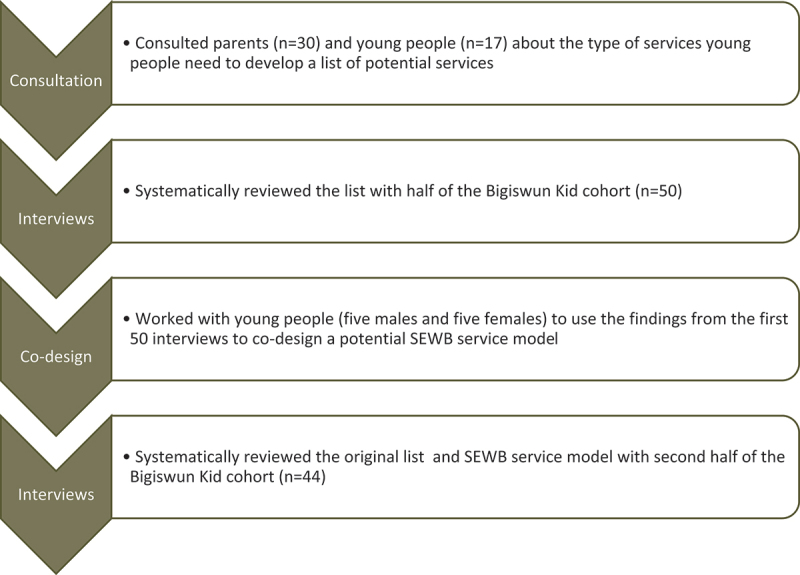
SEWB service co-design data collection process.

Halfway through the interviews with young people (*n* = 50), we worked with ten young people (five females and five males) to workshop the design of a SEWB service. The young people were consulted over several sessions in gender-specific groups. The group discussed common responses from the first 50 interviews regarding young people’s experiences accessing services within and outside the Fitzroy Valley, as well as their experiences with the support provided by the Bigiswun Kid Team so far. This process is outlined in Figure 1.

We focused on the types of support that should be provided through a SEWB service, the staff and the infrastructure required. A model was developed for service and staff, and these were discussed in the remaining interviews with young people (*n* = 44).

#### Learnings about engaging and partnering with young people

In this population-based study, we conducted interviews and provided support to young people with varying levels of need, including some of the most disengaged young people residing in the Fitzroy Valley. In the Supplementary Material, we describe what we learned about how best to support young people based on feedback from the young people in the Bigiswun Kid cohort and the research team’s experience.

### Statistical analysis

#### Quantitative

We calculated the frequency of young people and parents who requested each support.

#### Qualitative

The qualitative data were analysed using codebook thematic analysis (29). This approach was chosen as it enabled us to collate direct responses to specified questions: whether participants thought the listed support was needed, how they thought it should be delivered, and if they could think of any other support required. Hereafter, these are termed topics (Ayre & McCaffery, 2022). We employed an experiential approach, where the young people and their parents were viewed as experts whose views accurately reflected their experiences and needs (Braun & Clarke, 2022). Codebook thematic analysis recognises that researcher bias is inevitable in qualitative analysis and should be used as a tool (Ayre & McCaffery, 2022; Braun & Clarke, 2022). For this reason, we ensured that the team who interviewed the young people and worked with them to pilot the supports conducted the qualitative analysis. We believed that their experience working alongside young people through the Project, combined with their lived experience of coming from or working in the region, would inform their understanding and interpretation of the qualitative data. The four investigators (L.R., N.R., M.B., E.B.) who conducted the most interviews discussed the qualitative responses and support piloted to identify topics and learnings. During these discussions, the Aboriginal researchers (M.B. and E.B.) were acknowledged as having the most profound understanding, and therefore, their views were given priority. As the study was population-based, we had a large sample size for the qualitative data, which ensured saturation. Given the large sample size, common responses were easily identified as most participants repeated them. A non-Indigenous researcher (L.R.) drafted the topics and learnings, which were then reviewed by an Aboriginal researcher (E.B.) to ensure they accurately reflected the intended descriptions by the young people and their parents. Two Aboriginal chief investigators (E.C. and J.D.) reviewed the final summaries and provided additional context to recommendations to ensure they were adequately represented and understood.

## Results

### Participant demographics

In 2020 and 2022, 113 young people born between 2002 and 2003 resided in the Fitzroy Valley. Of these, 94 (83%) young people and 101 (88%) of their parents consented to participate in the interviews. Participant demographics are provided in Table 1.

**Table 1.** Participant demographics.

Demographic variables	Proportion/Mean (SD)
Frequency Age	Young people (*n* = 94); Parents (*n* = 101) 17.88 (0.73) range 16–19 years
Sex	46% Female, 54% Males
Primary caregiver	74% mothers, 6% fathers, 17% grandparents, or 3% others
Aboriginal	100% young people, 99% parents
Diagnosed with FASD*	22%
**Community location from town**	
Less than one hour from town	50%
One to two hours from town	50%

*Most of these diagnoses were made in the Lililwan Project, a community-led prevalence study.

### Mapping of existing mental health services

During data collection, two mental health services were available: the WA Country Health Service (WACHS) Child and Adolescent Mental Health Service (CAMHS) (0–17 years) and Kimberley Mental Health and Drug and Alcohol service (KMHDS) (for adults 18 years+), and a private drive-in, drive-out counselling service. Each WACHS service included one mental health nurse/social worker based in Fitzroy Crossing who worked alongside one female and one male Aboriginal mental health worker. Psychiatrists visited either monthly (for adults) or three or four times a year (for children and adolescents). According to the WACHS website, these services are available to people with moderate to severe mental health problems (Government of Western Australia WA Country Health Service, 2024). However, the experience of the Bigiswun Kid Team and young people was that, due to the high level of need, these services were only accessible to people with severe mental illness. The private service was designed to offer support to people with mild mental health needs. Depending on staff availability, they visited the Fitzroy Valley on a fortnightly to monthly basis during the dry season. However, staff turnover was high. With the change in staff came a change in qualifications and ways the staff worked with young people. During the two years of data collection, the counsellor role was filled by two social workers, an art therapist, and a clinical psychologist.

### SEWB service supports

**Figure 2.**
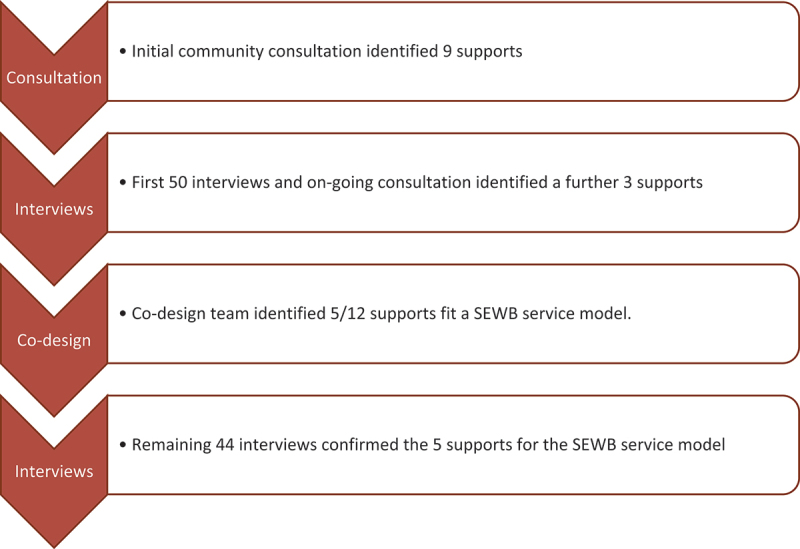
SEWB service co-design data collection process results.

During the consultation process, young people and their parents identified nine supports, which were reviewed in the interviews. An additional three supports were suggested during the interviews and continued consultation. Through service co-design consultations with young people, five of the 12 supports were selected to be included in a SEWB service; this process is illustrated in Figure 2. The five SEWB supports are outlined in Table 2 and discussed in more detail below. The remaining seven of the 12 supports will be described elsewhere. We piloted three of the five SEWB supports. Piloting formal mental health support was not feasible or appropriate, as it would not have remained accessible without ongoing funding after the research. Unfortunately, we were unable to identify a suitable facilitator to pilot men’s wellbeing workshops. Nevertheless, the young men did participate in weekly on-Country activities with the Bigiswun Kid Team.

**Table 2.** Proportion of young people and parents who requested each support and details of the piloted supports.

Support	Proportion of young people requesting support (n = 94)	Proportion of parents requesting support (n = 101)	Support piloted
1. Culturally appropriate mental health support e.g., psychology	100%	100%	-
2. Community wellbeing workshops*	-	-	Two suicide prevention workshops (19 Aboriginal women) Community wellbeing camp (60 people)
3. Women’s wellbeing workshops	95%	100%	Weekly women’s art therapy (40 people)
4. Men’s wellbeing workshops	72%	98%	-
5. Support navigating existing services	86%	89%	Supported young people to navigate existing services (84 people)

*These supports were not identified in the community consultation but identified by several young people and parents during the interviews. Hence, they were not systematically discussed with all interviewees, so these data are unavailable.

## Culturally appropriate mental health support

***Young people***. All 100% of young people wanted access to culturally appropriate mental health support. Below, we describe the topics that arose when discussing how mental health support could be delivered.

*Topic 1. Staff demographics*. Young people varied in who they would like to speak to about their feelings. The most common remark was that the service must employ local Aboriginal people as mentors for young people. Some requested that local Aboriginal people be trained as mentors to provide mental health/wellbeing strategies to young people and teach their parents and other older family members how to support the young people in implementing these strategies. The young people explained that Aboriginal mentors would ideally be known in their community for helping young people:

Someone we know, family or someone from our language group who can run programs around drugs, alcohol and mental health – young person

Certain people who young people can talk to, who follow up on you; it could be family or friends. It is hard to get help. – young person

However, other young people said that they would prefer to talk about their problems to an outsider not related to people in the Fitzroy Valley, as this felt more private and confidential:

It should be by someone who is not a friend or family and who is not related to the community – young person

An outsider who doesn’t know us, who we can trust – young person

It has to be a professional, so you know it’s confidential – young person

Young people who had previously accessed mental health services spoke of the importance of health providers being appropriately qualified. Young people who had accessed several services, usually one or two within the Kimberley and one or two in cities, said they felt most understood by, and got the most out of, speaking with a clinical psychologist rather than a counsellor or social worker:

The psychologist (in Perth) was the first person who got me, what was going on, and could help me understand – young person

I got more from one session with her (a clinical psychologist in Perth) than six sessions with the person up here – young person

These young people had complex lives, so the more specialised support offered by clinical psychologists was likely best suited to their needs.

Young people wanted someone available every week who would hold the role for at least 12 months. They felt that the sessions needed to be more frequent than once a month, even every fortnight, to be beneficial, and this was a common reason they disengaged from existing drive-in/drive-out services. Also, they did not want to repeat their problems with a new person, a common experience due to the high staff turnover in existing services:

I only wanna to tell my story once – young person

They (counsellor) only came once a month, what are we meant to do the rest of the time? It’s too long to wait – young person

*Topic 2. On-Country wrapped around an activity*. The young people explained that they would find it easier to talk about their feelings if it was wrapped around an activity, such as music or art, conducted on-Country or in their community. Having an opportunity to spend time on-Country was considered an essential part of a culturally appropriate mental health service. Some young people liked talking to someone while going for a walk or a “cruise”. A drive provides a quiet, private, air-conditioned space, which can be hard to find in remote communities with limited resources. A walk or drive also provides a sense of being connected with Country:

I was so scared to see the school counsellor. I didn’t want to do it and was stressing but she asked if I wanted to go for a walk, then I could talk - young person

*Topic 3. Group or individual sessions*. While some young people expressed a preference for group settings, others preferred individual sessions. We piloted the group format through art therapy. We found it helpful for engaging young people, sharing general information and strategies, and providing a stepping stone to individual mental health support for those who needed it. See the Supplementary Material for details.

***Parents.*** All 100% of parents said culturally appropriate mental health support was needed; many felt it was the most important service. Many parents felt more education was needed for young people and parents to help people understand their wellbeing:

Educate our people on mental health. About having a healthy mind and what makes you feel like that. Education and knowing who to talk to. I used to call it (suicide) selfish, but with training in mental health, now I have more empathy. - parent

Parents spoke of the importance of culturally appropriate services, which were best achieved by ensuring they were Aboriginal-led and designed in consultation with young people.

f it is run by MWRC … .people who know these kids and how to talk to them the right way- parent

Like the young people, some parents spoke of the need for qualified staff, adequately trained to support young people with complex mental health needs:

“Young people comfortable talking about stuff go to these services and should be right. Need staff with experience” – parent

One person emphasised the importance of services working together or having a service that helps connect to other services, such as police, justice, health, and education, so that the supports provided to the young person are coordinated:

“Not everyone has parents who can talk to them. We need partnerships with services working together” – parent

### Community wellbeing workshops/camps

The need for community wellbeing camps was not initially identified in the community consultation, but was suggested by parents during interviews and consultations with senior community members throughout the Project. Thus, we could not ask each person interviewed whether this was necessary and how it should be delivered. The Bigiswun Kid Team collaborated with a cluster of communities to pilot a community wellbeing camp and partnered with local school psychologists to provide two community suicide prevention workshops. When designing the SEWB service model with young people, they chose to incorporate community wellbeing workshops. Details about the community wellbeing camp and workshops, as well as the lessons learned from these, are described in the Supplementary Material. The following is an example of one such lesson.

People in the Fitzroy Valley are resilient and hopeful. These positive attributes stem from their connection to culture, Country, and kinship; the vision and motivation of their strong Aboriginal leaders; and the hope for and from their young people.

### Women’s wellbeing workshops

Almost all (95%) of the young women and 100% of their parents wanted wellbeing workshops for young women. These were described as opportunities for young women to connect with each other, with Country and culture, and learn about wellbeing concepts, strategies and services. The most common feedback was that these workshops should be run in communities and led or co-led by female mentors or other young women from each community:

Has to be run in (community name) or by the river- young person

(I would attend) “If it’s in a group and I’m not on my own – young person

This was important for several reasons. First, most people in Aboriginal communities in the Fitzroy Valley are related, and young people spend most of their time with other young people from their community, creating a safe space. Second, some young people do not leave their community when they are struggling, such as with mental health concerns. Third, having in community supports makes them accessible. People can come and go as they want and do not have to wait for a lift in and out of town (there is no public transport, and few people own a car). The location for initial workshops was important as it meant young people anxious about attending knew they could go home for a break when needed.

See the Supplementary Material for information about how we piloted weekly women’s art therapy for nine months and the lessons learned.

### Men’s workshops

Three-quarters (72%) of the young men and almost all (98%) of the parents wanted wellbeing workshops for young men. The young men wanted these programs run on-Country, in small groups, in individual communities, led or co-led by local Aboriginal men:

I would do this if (Aboriginal mentor) run’s it – young person

Parents thought men’s wellbeing programs should teach men how to manage their feelings. One parent who had attended a men’s workshop felt the workshops shouldn’t involve much talking at the start to help engage young men.

“Instead, they should take men on-Country and let them heal by doing things together. Upskill them without focusing on everything they do wrong. Out bush, we feel free and strong. Show them what to do rather than talking about what not to do”. – parent

### Support navigating and accessing existing services

Most (86%) young people and parents (89%) wanted a service that helps young people navigate and attend existing services. The most common services people wanted help navigating were:

1. Department of Transport (through the Shire) to obtain a driver’s licence. In turn, they needed support to navigate the services that provide the identification required to apply for a driver’s licence. e.g., birth certificate (Registry of birth, deaths, and marriages – visits the Fitzroy Valley a few times a year or apply remotely), bank card (closest is 250 km, so must apply remotely), Medicare card (apply remotely).
2. Employment service (local), tertiary education (remotely) or Centrelink (local) if no employment opportunities are available.
3. Health services include general practitioners, mental health, sexual health, dental care, and antenatal care (available through community clinics, Fitzroy Crossing Hospital, or by travelling to Derby 250 km, Broome 400 km or Perth 2,500 km).

Many of these services are not available locally, requiring young people to navigate complex forms and processes.

See the Supplementary Material for information on how we piloted supporting 84 young people in navigating existing services.

### SEWB service staffing model

During the SEWB service co-design consultation, the young people and Bigiswun Kid Team developed an optimal staffing model outlined in Table 3.

**Table 3.** SEWB service staffing model.

Staff	Role Description
Local Aboriginal mentors (at least one male and one female)	Mentors who can help young people navigate existing services and take them on-Country in groups or one-on-one to chat and learn wellbeing strategies, connect with the Country and find respite from daily life/overcrowding.
Social workers/counsellors (at least one male and one female)	Social workers who can help young people identify their goals and support them in achieving them, including navigating complex services and scaffolding support according to their needs.
Team leader	An older team leader who can help the team (likely comprising younger staff) build rapport and connections with Senior Aboriginal community members and senior staff at other local services. Having the support of senior community members is crucial to success in remote communities. The team leader would also help staff overcome challenges, supports capacity building and strategies to minimise burnout, and provide ongoing mentorship. This role could be divided into two: a cultural lead and a clinical lead.
Clinical psychologists	Clinical psychologists or similar who can provide therapeutic support. People’s views varied on whether this support should be provided through the proposed Bigiswun Kid SEWB Service or the existing WACHS mental health services (CAMHS and KMHDS). It was agreed that if the latter was preferred, efforts would need to be made to reduce the existing stigma associated with use of these services. If consistent clinical psychologists could only be provided via telehealth, the young people wanted to be supported by the local Bigiswun Kid Team to access telehealth. This would mean collecting the young people and driving them to a private place for the appointment, assisting with the telehealth technology, being available after sessions to take them to sit by the river to decompress, providing support to implement ongoing strategies and upskilling family members about how to assist with the strategies between sessions. The young people wanted the telehealth clinical psychologists to be the same person for all their sessions to ensure continuity of service and prevent the need to repeat the young person’s experience. Young people also wanted the telehealth psychologists to visit the Fitzroy Valley so they could understand what life is like for them and be better placed to provide feasible strategies for the geographic and cultural context.

The staffing model was reviewed by the second half of the young people (*n* = 44) who had not yet been interviewed and was well received. The most common feedback from people living in remote communities outside of Fitzroy Crossing was that the support needed to be accessible in remote communities. It was suggested that the primary team (as outlined in Table 3) be based in Fitzroy Crossing and that additional mentors be located in remote communities to run activities for young people and connect them to services and the town-based SEWB team as needed. Regarding the delivery of clinical psychology services via telehealth, some young people suggested that it would be best if it could be offered in a shared community space where they feel comfortable. Another typical response from young people is that they should be employed to assist with administration or help the mentors ensure they are guiding the service, allowing them to build their capacity to perform these roles in the future.

## Discussion

The first aim of this study was to engage young people from the Fitzroy Valley and their parents in identifying young people’s support needs to inform the development of a place-based SEWB service. Five supports were identified: culturally appropriate mental health support, community wellbeing workshops, male and female-specific wellbeing workshops, and support to navigate existing services. All young people and their parents requested mental health supports, and parents identified this as the priority for young people. The need for culturally appropriate mental health support aligns with previous research (Dudgeon et al., 2012, 2015, 2023; McPhee et al., 2022) and recommendations from government inquiries (Fogliani, 2019) in the Kimberley. The wellbeing workshops (for community, females, and males) provide opportunities for young people to connect with their family, community, Country, and culture, and participate in activities that make them feel good. Workshops enable the sharing of information about SEWB, including SEWB concepts, strategies, and available supports. This is consistent with four of the core elements of the Aboriginal and Torres Strait Islander model of SEWB: connecting with family, community, culture, and Country (Gee et al., 2014). Empowering young people to develop wellbeing (health and mental health) literacy is one of the World Health Organization’s global standards for quality adolescent health care (World Health Organisation, 2015). Finally, the request to integrate support for navigating all existing services into a SEWB service model reflects the holistic view of SEWB for Aboriginal and Torres Strait Islander people, encompassing mental, physical, cultural, and spiritual health (Gee et al., 2014). The need for this support also reflects the difficulty young people experience accessing services in very remote regions (Warwick et al., 2019).

The study’s second aim was to identify how young people would like the five SEWB supports delivered and how the service should be staffed. Through consultation with young people, it was decided that the SEWB service required four types of staff (male and female Aboriginal SEWB mentors, male and female social workers/counsellors, clinical psychologists, and a team leader who partners with and ensures senior community members guide the team). The need for administrative staff and additional Aboriginal SEWB mentors based in remote communities was later identified through interviewing the second half of the cohort, and while piloting supports. Having mentors in remote communities helps overcome the challenge of servicing a large geographical area, ensuring that young people are supported by someone well-known to and trusted by them. The involvement of local Aboriginal representatives from each community also provides the service with strong cultural governance, ensuring that the unique cultural differences of each community are considered and relevant protocols applied. The staffing model proposed is similar to that outlined in the Western Australian Aboriginal Community-Controlled Health Services (ACCHS) Social and Emotional Wellbeing Service Model. The ACCHS model proposes that staffing is adapted to suit the needs of each location and that a range of other services could be included, e.g., clinical psychologists (Aboriginal Health Council of Western Australia, 2021).

The recommendation for including clinical psychologists in the Bigiswun Kid SEWB Service model arose from consultations with young people about their experiences accessing mental health services, where they identified gaps, and a review of the available services. At the time of data collection, some services were accessible to people with mild mental health concerns (e.g., acute life stressors) and severe mental illness (e.g., attempted suicide, acute schizophrenia). However, there was a gap for people with moderate mental health concerns (e.g., complex trauma, post-traumatic stress disorder, persistent anxiety or depression). Based on the experiences of young people (both within and outside the Kimberley), a clinical psychologist can best fill this gap and address their needs. This finding is consistent with the views of health professionals surveyed throughout Australia who identified a “missing middle”, which was described as a gap in mental health services for people with moderate mental health needs who were not in crisis but required intensive and ongoing treatment and therapy (Petrie et al., 2021). If moderate mental health needs in the Fitzroy Valley are the remit of WACHS, as stated on their website (Government of Western Australia WA Country Health Service, 2024), but are inaccessible, this may reflect the incapacity of services to meet demand. It also demonstrates WACHS’s need for improved delivery of consistent, accessible clinical psychology services in the Kimberley, as highlighted in a recent review of Child Development Services (Legislative Council, 2024). Given the difficulty of recruiting and retaining staff in very remote regions for the long term, the team discussed with young people the possibility of accessing clinical psychology services, or equivalent, via telehealth. As described in the results, the young people provided a model for how this might work. A strength of both the proposed Bigiswun Kid and ACCHS SEWB service models is that, in conjunction with health services, they offer a culturally appropriate stepped-care model as outlined in the Framework (Aboriginal Health Council of Western Australia, 2021; Government of Western Australia WA Country Health Service, 2024).

The SEWB service design included three types of support (help navigating existing services, wellbeing workshops and formal mental health support). Based on piloting some of these supports, we found that this model helped young people prepare to seek professional mental health support. First, people typically sought help navigating existing services, as this was the most practical and least intimidating support. Assisting people to navigate services provides opportunities for the young people to develop connections with the staff and learn how the team works, which, when done right, encourages young people to ask for help with more serious issues, e.g., formal mental health support, sexual health services, and family and domestic violence services. Second, the workshops provided a space for young people to learn about and discuss topics of concern, as well as concepts related to wellbeing and mental health, the types of support available, and what they entail (e.g., what counselling, art therapy, and psychology entail). Developing an understanding of SEWB and the support available further prepared young people to seek formal mental health support. This stepped-care engagement process may be helpful for other communities implementing SEWB services or struggling to engage young people in mental health services.

### Limitations

Our research provides information for a place-based SEWB service design, which means the findings cannot be directly translated to all remote Aboriginal communities. However, many of our findings align with research conducted in other regions and could be helpful to different communities. This study aimed to collaborate with young people and their families to develop an initial plan for a SEWB service model. Future research is needed to build on this initial design and implement and evaluate a SEWB service for young people, which is currently underway. Culturally appropriate formal mental health support, such as clinical psychology, was the most requested SEWB support by parents and young people. A limitation of this study is that it was beyond the scope of the Bigiswun Kid Project to pilot formal mental health support. Therefore, an essential next step is to identify the most effective way to implement this support (e.g., through WACHS or the Bigiswun Kid Project, via telehealth or in-person) and evaluate its implementation, efficacy, and sustainability.

## Conclusion

In this Project, we identified the types of support young people needed within a SEWB service in the Fitzroy Valley and how they wanted them delivered. The need for place-based service design was highlighted through our consultations with young people and their parents. A place-based design ensured the supports were designed to meet the unique strengths and challenges of the region and each community. We also identified strategies for engaging and supporting young people in remote Aboriginal communities, which can be applied to all services supporting young people in the Fitzroy Valley and may also apply to some other remote Aboriginal communities. Through piloting these supports (see Supplementary Material), we demonstrated the importance of connecting with Country for promoting wellbeing and empowering communities and families to lead wellbeing initiatives. The support provided tangible examples of how SEWB services can foster these connections with Country, community and kinship.

The Bigiswun Kid Project Leadership Team utilised these findings to successfully secure funding from the WA Mental Health Commission for MWRC to implement and pilot a formal SEWB service for young people, known as the Bigiswun Kid SEWB Service. This is an excellent outcome for the community and critical first steps to improving SEWB for young people in the region. However, as Senior Aboriginal women noted in the suicide prevention workshops, systemic issues must be addressed if we are to improve the SEWB of all people in the Fitzroy Valley. While a SEWB service will provide selective interventions, a broader universal response that addresses the systemic risk factors for distress and mental ill-health is also needed (Dudgeon et al., 2016).

## Supplementary Material

Consider statement for SEWB CoDesign

Supplementary Material Bigiswun Kid Project SEWB Service CoDesign

COREQ Checklist Bigiswun SEWB Service Design

## Data Availability

MWRC, on behalf of the Fitzroy Valley Community, is the custodian of the data collected, and the knowledge generated. The data are stored at the University of Sydney for security and confidentiality reasons. The information collected in the community may be used in future research with MWRC’s approval. Requests to access the data can be made to Dr Lauren Rice (lauren.rice@sydney.edu.au) or Prof Elizabeth Elliott (elizabeth.elliott@health.nsw.gov.au).

## Note

1. Consistent with Western Australian Government reports, in this article, we use the term Aboriginal people in preference to “Aboriginal and Torres Strait Islander”, recognising that Aboriginal people are the original inhabitants of Western Australia.

## Partnership

The roles and expectations of partners (MWRC and the University of Sydney) were outlined in a description of how we work together, which has evolved since the partnership began in 2008, along with a Values and Ethics Statement. A formal Research Agreement was also established to outline how funds would be shared between the two partners. A risk matrix was developed at the start of the project to identify potential project risks and strategies for minimising risk, which was reviewed throughout the project.
