# Supplementary material for: Co-designing a place-based social and emotional wellbeing service model with young Aboriginal people in the remote Fitzroy Valley of Western Australia: the Bigiswun Kid project
Source: Aust J Psychol. 2025 Aug 3;77(1):2538509. doi: 10.1080/00049530.2025.2538509 (PMC12320268; doi:10.1080/00049530.2025.2538509)
Supplement: Consider statement for SEWB CoDesign [file RAUP_A_2538509_SM3011.docx]

| Governance | |  |
| --- | --- | --- |
|  | | *Specify how and where you’ve addressed this in the paper, or note ‘Not applicable’ and provide reasoning.* |
| 1. | Describe partnership agreements between the research institution and Indigenous-governing organisation for the research (e.g., informal agreements through to MOU (Memorandum of Understanding) or MOA (Memorandum of Agreement)). | **Page 28 under Partnerships states:** “The roles and expectations of partners (MWRC and The University of Sydney) were outlined in a description of how we work together, which has evolved since the partnership began in 2008, and a Values and Ethics Statement. A formal Research Agreement was also established to outline how funds would be shared between the two partners.” |
| 2. | Describe accountability and review mechanisms within the partnership agreement that addresses harm minimisation. | **Page 28 under Partnerships states:** “A risk matrix was developed to identify potential project risks and strategies for minimising risk, which was reviewed throughout the project.” |
| 3. | Specify how the research partnership agreement includes protection of Indigenous intellectual property and knowledge arising from the research, including financial and intellectual benefits generated (e.g., development of traditional medicines for commercial purposes or supporting the Indigenous community to develop commercialisation proposals generated from the research). | **Page 28 under Data availability Statement states:** “MWRC, on behalf of the Fitzroy Valley Community, is the custodian of the data collected, and knowledge generated.” |
| Prioritisation | |  |
| 4. | Explain how the research aims emerged from priorities identified by Indigenous stakeholders, governing bodies, funders, non-government organisation(s), stakeholders, consumers and empirical evidence. | **The middle paragraph on page 8 states:** “In 2019, they (senior Aboriginal women) initiated the Bigiswun Kid (Kimberley Kriol for adolescent) Project to consult the young people from the Lililwan Project about the supports they need to thrive in adolescence and early adulthood (Rice et al., 2022)……. The women wanted to focus on older adolescents (16-19 years) because they recognised a service gap for young people and a need to support them in transitioning out of school”. |
| Relationships (Indigenous stakeholders/participants and research team) | |  |
| 5. | Specify measures that adhere and honour Indigenous ethical guidelines, processes and approvals for all relevant Indigenous stakeholders, recognising that multiple Indigenous partners may be involved, e.g., Indigenous ethics committee approval, regional/national ethics approval processes. | **Page 29 under Ethics approval and consent to participate states:** “This study was reviewed by the Kimberley Aboriginal Health Planning Forum Research Subcommittee and received ethics approval from the Western Australian Aboriginal Human Research Ethics Committee (#914) and the Western Australian Country Health Ethics Committee (#RGS3424). A Values and Ethics Statement was developed for the project which was prepared in line with the following three documents: Ethical conduct in research with Aboriginal and Torres Strait Islander Peoples and communities: Guidelines for researchers and stakeholders (NHMRC 2018); Keeping Research On Track II: A companion document to Ethical conduct in research with Aboriginal and Torres Strait Islander Peoples and communities: Guidelines for researchers and stakeholders (NHMRC 2018): and the National Statement on Ethical Conduct in Human Research (NHMRC / AVCC 2007).” |
| 6. | Report how Indigenous stakeholders were involved in the research processes (i.e., research design, funding, implementation, analysis, dissemination/recruitment). | **Page 9 under Positionality states:** “ This study was part of a 16-year partnership between Senior Aboriginal women from the Fitzroy Valley, currently represented by MWRC (E.C.), and clinician researchers (E.J.E.) from the University of Sydney [13]. The authors include Aboriginal researchers from the Fitzroy Valley (E.B., M.B., C.C., J.D., E.C.), non-Indigenous researchers (L.R., E.J.E., S.T.) and youth mentors (N.R.., F.W.). Non-Indigenous staff received formal cultural awareness training and ongoing cultural supervision and guidance.  **Page 29 under Author Contribution states** (Aboriginal researchers in bold): “Conceptualisation, **E.C., J.D.** and E.E; methodology, **E.C., J.D., E.B., M.B**., L.R, and E.E.; formal analysis, L.R, **E.B., M.B**., and NR; investigation, **E.C., E.B., M.B., C.C., J.D.**, NR and L.R.; resources, S.T; data curation, NR, and L.R.; writing—original draft preparation, L.R..; writing—review and editing, **E.E., E.B., E.C., J.D**.; supervision, **E.C.**, E.E., L.R., and S.T; project administration, L.R.; funding acquisition, **E.C**., L.R., E.E. and S.T. All authors have read and agreed to the published version of the manuscript.”  **Page 10 under Community Consultation states:** “We conducted an 18-month community consultation, during which we spoke to young people (n=17), parents (n=30), and stakeholders (n=23), e.g., senior community members, local government services, and other ACCOs, about the purpose and design of the Bigiswun Kid Project. .”  **Page 10 under interviewers states:** The interviewers included two full-time Aboriginal researchers/youth mentors (EB, MB) from the Fitzroy Valley, a non-Indigenous Research Fellow (LR), and two non-Indigenous youth mentors (NR, FW). |
| 7. | Describe the expertise of the research team in Indigenous health and research. | **See page 2 of Title Page and Biographies for full list of author biographies.** |
| Methodologies | |  |
| 8. | Describe the methodological approach of the research including a rationale of methods used and implication for Indigenous stakeholders, e.g., privacy and confidentiality (individual and collective) | **Page 10 under Project design states:** The Bigiswun Kid Project is a population-based study that used an Aboriginal Participatory Research Action (APAR) approach. For details about the methods, see our protocol paper (Rice et al., 2022) and a paper describing the APAR approach (Rice et al., Accepted), including Aboriginal leadership and governance.  **Page 28 under Data availability statement states:** “The data are stored at The University of Sydney for security and confidentiality reasons.” |
| 9. | Describe how the research methodology incorporated consideration of the physical, social, economic and cultural environment of the participants and prospective participants (e.g., impacts of colonisation, racism and social justice), as well as Indigenous worldviews. | **Page 11 under Interviewers states:** Eleven Aboriginal community navigators were also employed to provide the team with community-specific knowledge, language skills and cultural guidance and governance. They helped locate, inform and seek consent from parents and young people about the study, to support them during the study, and if needed, translate into traditional language. At least one navigator was engaged from each cluster of communities (based on distance from Fitzroy Crossing and proximity to each other) across the Fitzroy Valley.”  Also, see response to criterion 15 below. |
| Participation | |  |
| 10. | Specify how individual and collective consent was sought to conduct future analysis on collected samples and data (e.g., additional secondary analyses; third-parties accessing samples (genetic, tissue, blood) for further analyses). | **Page 28 under Data Availability Statement states: “**The information collected in the community may be used in future research with the approval of MWRC.” |
| 11. | Described how the resource demands (current and future) placed on Indigenous participants and communities involved in the research were identified and agreed upon, including any resourcing for participation, knowledge and expertise. | **Page 11 under Interviews:** “The young people and parents were provided with a $50 grocery store voucher to thank them for their time completing the interviews.”  **Page 10 under Community consultation states:** “During the consultation, senior community members requested that support be provided to young people during the data collection to ensure immediate knowledge translation and benefit of the research. We spoke with 30 parents and 17 young people to determine what type of supports young people would like, described below.”  **Page 12 under Piloting support in the research:** “Based on the support needs identified in the consultation and interviews and the gaps identified through mapping services, the Bigiswun Kid Team worked with the young people to determine the supports they could provide and pilot during the research. Extensive consultation with senior community members, parents, and young people continued throughout the Project, including when a support was piloted. The team kept notes from these consultations, which were used to develop the learnings for each support.” |
| 12. | Specify how biological tissue and other samples including data were stored, explaining the processes of removal from traditional lands, if done, and of disposal. | No samples were taken. |
| Capacity | |  |
| 13. | Explain how the research supported the development and maintenance of Indigenous research capacity (e.g., specific funding of Indigenous researchers). | **Page 11 under Interviewers states:** “The interviewers included two full-time Aboriginal researchers/youth mentors (E.B, M.B) from the Fitzroy Valley, a non-Indigenous Research Fellow (L.R), and two non-Indigenous youth mentors (N.R, F.W). Eleven Aboriginal community navigators were also employed…..Everyone working on the project received training in the research activities, including the protocol and confidentiality, and the two full-time staff were offered additional training opportunities, for more details see (Rice et al., Accepted).” |
| 14. | Discuss how the research team undertook professional development opportunities to develop the capacity to partner with Indigenous stakeholders. | **Page 9 under Positionality states:** “Non-Indigenous staff received formal cultural awareness training and ongoing cultural supervision and guidance. We used reflexive practices to consider how our lived experiences shaped our perspectives throughout the Project.” |
| Analysis and interpretation | |  |
| 15. | Specify how the research analysis and reporting supported critical inquiry and a strength-based approach that was inclusive of Indigenous values. | **Page 13/14 under Qualitative Data Analysis states: “**We used an experiential approach, where the young people and parents were seen as experts whose views accurately represented their experiences and needs (Braun and Clarke, 2022). Codebook and reflexive thematic analysis recognise that researcher bias is inevitable in qualitative analysis and should be used as a tool (Ayre and McCaffery, 2022; Braun and Clarke, 2022). For these reasons, we ensured that the team who interviewed the young people and worked with them to pilot the supports conducted the qualitative analysis. We believed their experience working alongside young people through the Project and their lived experience of coming from or working in the region would inform their understanding and interpretation of the qualitative data. The four investigators (L.R, N.R, M.B, E.B) who conducted the most interviews discussed the qualitative responses and supports piloted to identify topics and learnings. During these discussions, the Aboriginal researchers (M.B. and E.B.) from the region were acknowledged as having the most profound understanding and, therefore, their views were privileged. |
| Dissemination | |  |
| 16. | Describe the dissemination of the research findings to relevant Indigenous governing bodies and peoples. | Public dissemination is pending publication of these data so has not been reported in this manuscript. |
| 17. | Discuss the process for knowledge translation and implementation to support Indigenous advancement (e.g., research capacity, policy, investment). | **Page 26 under Conclusions states:** “The Bigiswun Kid Project Leadership team (MWRC leaders and USYD researchers) used the findings in this paper to successfully secure funding from the WA Mental Health Commission for MWRC to implement and pilot a formal SEWB service for young people, named the Bigiswun Kid SEWB Service. MWRC also secured funding for an independent formal evaluation of the service.” |
