# Supplementary material for: Co-designing a place-based social and emotional wellbeing service model with young Aboriginal people in the remote Fitzroy Valley of Western Australia: the Bigiswun Kid project
Source: Aust J Psychol. 2025 Aug 3;77(1):2538509. doi: 10.1080/00049530.2025.2538509 (PMC12320268; doi:10.1080/00049530.2025.2538509)
Supplement: Supplementary Material Bigiswun Kid Project SEWB Service CoDesign [file RAUP_A_2538509_SM3010.docx]

**Community-led place-based design of a social and emotional wellbeing service for young Aboriginal people in the Fitzroy Valley: Supplementary Material**

The following information is provided in the supplementary material.

1. We have summarised the following three supports piloted during the research, as well as the lessons learned from these pilots. The lessons were identified by reviewing notes taken during consultations with young people, their families, and senior community members, as well as after each support was piloted.
   1. Community wellbeing workshops/camps (community wellbeing camp and suicide prevention workshops)
   2. Women’s wellbeing (art therapy) workshops
   3. Navigating and accessing existing services
2. Common themes found across lessons learned.
3. The design of a SEWB service building.
4. Strategies for engaging young people in the Fitzroy Valley.

**1. Support piloted and lessons learned**

**a. Community wellbeing workshops/camps**

Support piloted - Community wellbeing Camp: This camp was initiated, designed, and led by senior community members to bring people from four surrounding communities together with local services to discuss and learn strategies for supporting young people’s social, emotional, and wellbeing (SEWB), with a focus on emotion regulation, as chosen by the community. The senior community members decided who from the community they would ask to organise workshops and activities during the camp and what resources were needed. The community members asked the Bigiswun Kid Project Team to identify and invite services that support young people in the region so that they could inform young people and their families about these programs. The community members also provided a list of needed resources, and the Bigiswun Kid Project Team collaborated with local services to gather these resources (e.g., 4WDs, camping supplies, food, art supplies, and tool-making materials). The camp included 60 community members and staff from four services. More community members wanted to join, but there was only enough car space for 60 community members plus staff. More services also wanted to join, but the camp was run during school holidays when services were short-staffed due to a lack of school holiday programs in remote regions. The senior community members chose to run it during the school holidays so more community members could attend. The camp ran over three days and included various activities that senior community members used to discuss emotion regulation strategies. E.g., learning patience through carving, expressing feelings and managing stress through art, and connecting to culture and kinship through damper and dance competitions. Visits were also made to cultural sites, where elders and senior community members explained the history and cultural significance of the sites. At the Marninwarntikura Women’s Resource Centre annual general meeting, a senior community member who attended the camp said it was the best thing that happened in their community that year—news about the camp’s success spread, leading to other communities requesting a similar collaboration.

Learnings from the community wellbeing camp

1. **Community wellbeing camps strengthen connections to family, culture, kinship, and Country**. Being on-Country provided a healing environment where participants could reconnect with Country, participate in cultural activities and connect with one another. The connection to Country is deeply ingrained in cultural identity, offering a sense of belonging. Participants often expressed how being on-Country helped alleviate the stresses of daily life. One young person remarked, "When I go out bush, I can breathe better.”
2. **Community wellbeing camps help shift the power balance from services to communities.** This shift empowers Aboriginal communities and strengthens community engagement and collaboration with services. The camp allowed community members and staff from local services to connect, learn from each other, share stories, and foster partnerships that continued beyond the camps.
3. **Community wellbeing camps provide a safe space for knowledge sharing.** These events highlight the effectiveness of workshops and on-Country camps for community knowledge sharing, particularly compared to meetings held in halls or offices. On-Country workshops and camps are underappreciated and underutilised by services. They provide community members with space away from daily life and overcrowded living to think about and discuss important issues. They also offer opportunities for older community members to pass on knowledge to the young.

**Support piloted - Suicide prevention workshops:** During the interviews, when asked who they talk to when they feel suicidal, most young people said they speak to a parent or primary caregiver (grandparent, aunty, uncle). Some of the parents we interviewed said they don’t know how to respond when their child tells them they are suicidal. We learned that the Mental Health Commission provided funding to school psychologists to train professionals in the area, mostly teachers, in the Gatekeeper Suicide Prevention Training to help them feel prepared to respond when someone is suicidal. The Bigiswun Kid Project Team approached the school psychologists to inform them that young people preferred turning to a parent or family member over a professional for help. The school psychologists kindly agreed to work with our team to adapt the Gatekeeper Suicide Prevention Training for parents/caregivers in the Fitzroy Valley.

We conducted two workshops: the first aimed to discuss ways of modifying the Gatekeeper Suicide Prevention Training, and the second sought to apply this adapted approach to a cluster of communities. In workshop one, we brought three school psychologists together and nine senior Aboriginal women from across the Fitzroy Valley to modify the Gatekeeper Suicide Prevention Training for families in the Fitzroy Valley. The nine senior Aboriginal women were chosen for being the people young people turned to the most in their communities.

In workshop two, we brought together two school psychologists and ten Aboriginal women from a cluster of three communities. Initially, the plan was to try the adapted Gatekeeper Suicide Prevention Training approach; however, with community input, this evolved into a broader discussion about how these three communities can take action to address youth suicide. The women identified challenges that parents often faced when supporting young people who might feel suicidal, and together, they identified strategies for overcoming these challenges. The challenges included social media, responding to threats of suicide, what to say in a crisis, a lack of resources, generational differences, adhering to cultural protocols, not all young people being ready for help, high exposure to suicide, and helping young people process grief. The women decided that strategies for addressing these challenges must occur at three levels: the community, family, and individual. Strategies were identified at each of these levels, which extended beyond just addressing suicide to improving the wellbeing of the community, family, and individual.

Learnings from the suicide prevention workshops

1. **People in the Fitzroy Valley are resilient and hopeful**. These positive attributes stem from their connection to culture, Country, and kinship; the vision and motivation of their strong Aboriginal leaders; and the hope for and from their young people.
2. People in the community want to know how to support their community members when they are suicidal and about mental health and wellbeing more broadly.
3. **Training should be solution-focused and give people clear, easy-to-remember steps for responding when someone feels suicidal.** For example, prioritise safety first, stay calm, and create a calming environment. Connect with the person and devise a plan to keep them safe.
4. **SEWB strategies should occur at three levels: the community, the family, and the individual.** Community-led workshops enable community members to collaborate and identify these strategies together. Community members spoke of the benefit of partnering with MWRC and the school psychologist, a local organisation, to co-facilitate the workshops.
5. **There needs to be greater access to psychologists and opportunities for people to spend time on-Country to heal**.
6. **Systemic change is required across all services**. While essential, these workshops will not address the root cause of suicide, which is the continued adversity caused by colonisation. Proper investment in co-designing community-led system change is required to empower communities to break the cycles caused by colonisation.

**b. Women’s wellbeing workshops**

**Support piloted – weekly art therapy:** Our team partnered with an art therapist employed by the Family and Domestic Violence Team at MWRC to run weekly on-Country art therapy sessions for young women aged 16 to 21 years. These sessions were held every Wednesday for two to three hours over a nine-month period. Each session was conducted either in community or on-Country. Attendance was strong, with 10 to 20 young women attending each session. Sessions began with an art therapy activity for the first hour, followed by several hours spent creating art, listening to music, fishing, or swimming. The art therapy activity was always optional. The art therapist provided some canvas, paints, or other mediums. The young women worked together to identify topics for each session, guided by the art therapist to encourage group discussion.

Learnings from the women’s wellbeing workshop

1. **Group workshops are a great way to engage young people and allow them to tailor support to their needs and at their own pace**. Before engaging the art therapist, the young women’s workshops were led by the female Bigiswun Kid Project Team members, who started by hiring a room at the local resource centre and providing a range of resources that young women said they liked, such as music, face masks, foot spas, make-up, art supplies, and cosy bean bags to chill out. These sessions attracted a few people popping in and out. However, it was typically younger girls (12 to 15 years). Eventually, some young women (16 to 21 years old) began attending and told the team they had been watching them run the sessions, but didn’t join immediately. They needed to see that the team was consistent and reliable to help build trust. Once five or six young women began attending the Bigiswun Kid Project Team worked with them to redesign the sessions. The young woman suggested doing it earlier in the day and picking up the young woman. They also requested that workshops be specific for young women aged 16 to 21 so that the young women did not feel they had to look after the younger girls and could talk freely. Over time, the sessions evolved from being a safe space for young women to hang out to more formal wellbeing workshops with the inclusion of the art therapist. This evolution was critical for engaging the young people and occurred as the young women got to know and build connections and trust with the Bigiswun Kid team. Even when the art therapist began leading the sessions, the workshop topics were still decided by the young women based on what was happening in their lives and their community. The young people and sometimes their mothers or other family members privately spoke with the Bigiswun Kid team to flag a situation or concern. The team would then work with the art therapist to discuss appropriate content for discussions, potential strategies to work on, and how to incorporate them to be culturally relevant, etc.
2. **Group workshops allow knowledge sharing and help familiarise young people with SEWB concepts.** The group format was a great way to explain art therapy and other forms of counselling and provide strategies and information about a broad range of topics. Some of the issues identified by young people and covered in the workshops included the importance of self-care, techniques to help regulate, connecting to Country and culture, learning different mediums to express and understand feelings, identifying strong people in the community to turn to for help, understanding the effects of drug and alcohol on health and mental health, identifying goals and mapping out the steps to achieve the goals, the importance of supporting each other and building each other up, especially during times of need.
3. **Group formats were often a necessary stepping stone to individual support for SEWB.** These sessions provided the young women with an understanding of counselling, which led some to use the free time at the end of each session to chat one-on-one with the art therapist or to book an individual session for another time. Other young women, those who thought they might have been struggling with a mental illness or stressful life event, expressed a desire to speak to a clinical psychologist. The team worked hard to identify a clinical psychologist. Unfortunately, there were none in the region or available through telehealth, so the team helped connect them to the local WA Country Health Service Mental Health Team.

**c. Navigating existing services**

**Support piloted:** We supported 84 young people to navigate various existing services. This could range from helping someone apply for a bank card to walking alongside young women as she attends each antenatal appointment throughout her pregnancy.

Learnings from supporting young people to navigate and access existing services

1. **Scaffolded support is essential.** Throughout the interviews, parents emphasised the importance of services, acknowledging that not everyone has the same skill set or understanding. Instead, services must get to know the person and adapt their approach to support that person accordingly. We found this essential to supporting young people. Scaffolding support involves providing tailored support to each young person and gradually removing it as they develop the necessary skills. This ensures young people are being met where they are and empowered to achieve as much as possible within their abilities. Supporting people in navigating services is a great way to build rapport and understand the type and level of support each young person needs, which can then be used to adapt future support.
2. **Role modelling and opportunities to ask questions. Role modelling is preferred to verbal instruction.** When role modelling, it is important to go slow and give the person time to watch what you’re doing. Encouraging people to ask questions, letting them know there is no wrong question and ensuring all questions are answered without judgement.
3. **Supporting people to access services can be an effective stepping stone to identifying and providing more intensive support.** Many young people we worked with first requested support to access an existing service, and through this process, they felt more comfortable discussing more sensitive support needs. For example, someone might come to request help to gather the ID needed for a driver’s licence, and once they get to know the team and develop trust and a connection, they begin to talk about their mental health needs

**2. Common themes that arose across the lessons learned piloting support**

A theme consistent across supports was the importance of empowering families and communities to be involved in, or lead, wellbeing initiatives. For example, when discussing culturally appropriate mental health supports, young people spoke of the need for local Aboriginal mentors who were trained to build the capacity of older family members to support young people’s wellbeing. We piloted this initiative by employing local Aboriginal people as mentors for young people and to assist with community-led suicide prevention workshops, where senior Aboriginal women reinforced the importance of building capacity to improve wellbeing, not only for the individual but also for their family and community. Empowering families and communities in wellbeing initiatives aligns with Principle Seven of the SEWB Framework, which recognises the importance of family, kinship and reciprocity in Aboriginal culture (1).

Another common theme was the importance of connecting with Country. At the request of senior community members, the community wellbeing camp, a community wellbeing workshop, and young women’s wellbeing workshops were all conducted on-Country. Where possible, on-country opportunities were provided to young people after they were supported in accessing existing services. From these activities, the Bigiswun Kid team saw firsthand how being on-Country helped young people decompress from daily life stressors. For example, young people spoke about how being on-Country allowed them to breathe better and relax. Through feedback from the camps and workshops, we learned that being on-Country provided opportunities for people to connect and come together, including community members and services, and provided safe places for discussions about wellbeing concepts and strategies. Consistent with our experience, previous studies have demonstrated the importance of Country to Aboriginal and Torres Strait Islander peoples’ health and wellbeing (2-6). SEWB services must be adequately funded to empower families and communities to be involved in wellbeing initiatives and provide on-Country opportunities, including workshops, camps, and one-on-one outings for young people.

**3. SEWB service building design**

The Bigiswun Team collaborated with young people to design a SEWB service building. This was initially designed by ten young people (five males and five females) and then reviewed in the remaining 44 interviews with young people.

During discussions with the ten young people, it was noted that a result of the Lililwan Project was that MWRC secured funding to establish the Baya Gawiy Children and Family Centre. The centre got its name as the structure is shaped like a Baya Gawiy (freshwater stingray). The structure is divided into two buildings; one side houses the Early Childhood Centre, and the other the Parent and Family Centre, which offers a range of family and child services, including playgroups, parenting programs, and visits from child health specialists, including the paediatric team. The young people thought there should be something similar for adolescents and young adults, ideally aged 17-25 years, where one side is a place for young people to spend time, with separate sections for males and females. The other side would house mentors and visiting services, including a sexual health nurse, a general practitioner (GP), and mental health professionals. They requested that it be located at the back of MWRC, so that young people could enter via the oval, a spot where they commonly hang out. They liked that it could look like they were going to the oval or SEWB chill out space, but could then pop in to see the sexual health nurse without everyone knowing. This was a concern, as many felt there was little privacy at the local hospital, where everyone could see you in the waiting room and observe which health service was calling you.

1. **Strategies for engaging young people**

Table 1 below outlines the factors that young people identified as most important for engaging young people in supports to foster SEWB. Table 2 outlines the factors the Bigiswun Kid Team found most important for engaging young people and their families based on their experience supporting young people through the research and piloting supports over two years.

**Table 1. The factors young people identified as being necessary for engagement**

| **Factor** | **Description** |
| --- | --- |
| **Be consistent** | The lack of Aboriginal people employed in government or private services, high staff turnover, short-term funding, and changes in policies, and processes results in lack of consistency of service provision, particularly services run by drive-in drive-out service. Consistency is a way services can build trust and is essential for building relationships with young people. Below are some strategies to ensure consistency suggested by young people.   1. Do what you say you will do, and do not promise anything you may not be able to provide. 2. Always be there when you say you will be. 3. Consistency of staff, particularly non-Indigenous staff, over time is the best way to gain the trust of a young person. When trust has been built, it is easier for staff to do their job effectively and efficiently. |
| **Be efficient** | Being efficient helps build rapport. To overcome mistrust, it helps to begin by supporting the young person with a small goal that can be quickly achieved (e.g., spending time on County or obtaining a bank card). This also allows staff to show they can and will help young people achieve their goals. When achieving a goal will take some time, staff should clearly explain why, how long the process will take, and what steps are involved. It can help to list the steps or provide a visual representation so that incremental progress can be seen through achieving each step. |
| **Be open-minded and only ask what is required** | All staff, Aboriginal or non-Indigenous, working with young people must be open-minded, non-judgemental, and willing to accept young people’s differences. Non-Indigenous staff must regularly reflect on their preconceived ideas of Aboriginal youth and how these might influence how they perceive and interact with young people. All staff should only ask the questions that are necessary for service provision and building rapport. Young people reported feeling uncomfortable by staff who ask many personal questions, especially when first meeting someone. |
| **Help breakdown challenges for young people** | Prioritise goals and support young people through tasks using a stepwise approach rather than expecting them to navigate things on their own. This is particularly important for accessing existing services, for example just dropping young people off to a service is inadequate and they need to be accompanied into the service until you know they can negotiate this on their own. |
| **Privacy and confidentiality** | Privacy and confidentiality are particularly important in very remote communities where people are well-known to each other. Young people requested personal conversations be conducted in private spaces, information kept by services is only accessible to those who require access and staff are trained not to share information outside of their workplace/role without the young people’s permission. |
| **Cultural safety** | Young people said they only felt comfortable engaging with non-Indigenous staff who respected their culture and way of life. |

**Table 2. The factors the Bigiswun Kid Team identified as necessary for young people based on their experience providing support for two years.**

| **Factor** | **Description** |
| --- | --- |
| **Aboriginal leadership** | Services for young people are most effective when they involve Aboriginal leadership and governance, ideally through an ACCO/Aboriginal Medical Service, to ensure it is embedded within the community, culturally safe and responsive and meeting community-identified need. |
| **Supports adapted for individual communities** | Having the support of senior community members and working with them to implement place-based support designed for specific communities or clusters of communities is essential for community engagement. As noted above, there is much inconsistency in services, particularly drive-in, drive-out, which has led to some mistrust. Young people were more willing to trust a support/service that had the support of their community leaders. Adapting supports for each community ensured their success, which further promoted engagement. |
| **Aboriginal mentors** | Employ Aboriginal mentors who the young people trust. |
| **Non-Indigenous staff willing to be guided by Aboriginal staff** | Non-Indigenous staff must be willing to be guided by Aboriginal staff and community members. |
| **Personal rapport** | Take the time to develop genuine rapport and connections with young people and obtain their trust. This typically involves a less formal relationship than in a city-based health setting. However, the staff felt this was achievable despite maintaining professional boundaries. |
| **Scaffolded support** | Offer tailored, scaffolded support that meets the needs of each young person and over time will help them achieve their goals and develop the skills to do things independently. |

**References**

1. Commonwealth of Australia. National Strategic Framework for Aboriginal and Torres Strait Islander Peoples’ Mental Health and Social and Emotional Wellbeing 2017–2023. In: Department of Prime Minister and Cabinet, editor. Canberra2017.

2. Butler TL, Anderson K, Garvey G, Cunningham J, Ratcliffe J, Tong A, et al. Aboriginal and Torres Strait Islander people's domains of wellbeing: A comprehensive literature review. Social science & medicine. 2019;233:138-57.

3. Dudgeon P, Derry KL, Mascall C, Ryder A. Understanding Aboriginal models of selfhood: The National Empowerment Project’s cultural, social, and emotional wellbeing program in Western Australia. International Journal of Environmental Research and Public Health. 2022;19(7):4078.

4. Fatima Y, Liu Y, Cleary A, Dean J, Smith V, King S, et al. Connecting the health of country with the health of people: application of" caring for country" in improving the social and emotional well-being of Indigenous people in Australia and New Zealand. The Lancet Regional Health–Western Pacific. 2023;31.

5. Terare M, Rawsthorne M. Country is yarning to me: Worldview, health and well-being amongst Australian First Nations people. The British Journal of Social Work. 2020;50(3):944-60.

6. Yap M, Yu E. Operationalising the capability approach: developing culturally relevant indicators of indigenous wellbeing–an Australian example. Ox Dev Stud. 2016;44(3):315-31.
